# Unilateral macular neovascularization formation during the follow-up of a 15-year-old boy with Bietti crystalline dystrophy and the successful treatment outcome with a single intravitreal ranibizumab injection

**DOI:** 10.3205/oc000214

**Published:** 2023-03-01

**Authors:** Mustafa Kayabaşi, Ferdane Ataş, Ali Osman Saatci

**Affiliations:** 1 Department of Ophthalmology, Dokuz Eylul University, Izmir, Turkey; 2 Department of Ophthalmology, Çerkezköy State Hospital, Tekirdağ, Turkey

**Keywords:** Bietti crystalline dystrophy, macula, macular neovascularization, optical coherence tomography, optical coherence tomography angiography

## Abstract

**Objective:**

To report the successful outcome with a single intravitreal ranibizumab injection in a 15-year-old boy with Bietti crystalline dystrophy (BCD) who developed a unilateral macular neovascularization (MNV)

**Methods:**

A retrospective case report

**Results:**

A 15-year-old Caucasian boy with Bietti crystalline dystrophy was diagnosed to have a unilateral MNV a year after the initial examination with the help of multimodal imaging and he was treated with a single intravitreal ranibizumab injection. Five months later, visual acuity improved and anatomically a dry-looking macula was noted.

**Conclusion:**

MNV is among the rare macular complications of BCD. The present case is the third reported pediatric case with MNV in association with BCD and the first pediatric BCD patient who received a vascular endothelial growth factor inhibitor (anti-VEGF) agent.

## Introduction

Bietti crystalline dystrophy (BCD) is a rare genetic disease characterized by the presence of retinal crystalline deposits mostly localized in the posterior pole and varying degrees of chorioretinal atrophy commencing at the central retina with or without accompanying perilimbal, subepithelial and anterior stromal corneal crystals [1], [2]. As the atrophic changes ensues, intraretinal crystals are likely to diminish in number [3]. Onset of the disease can occur from early teenage years to third decade of life. Some of the previously reported macular changes were cystoid macular edema [4], subfoveal serous retinal detachment [5], macular hole [6], and macular neovascular membrane (MNV) [7], [8], [9], [10], [11], [12], [13]. We hereby report the successful outcome with a single intravitreal ranibizumab injection in a 15-year-old boy who developed a unilateral MNV during the disease course who happened to be the third reported pediatric case with MNV in association with BCD and the first pediatric BCD patient receiving any vascular endothelial growth factor inhibitor (anti-VEGF) agent.

## Case description

**Figure 1:**
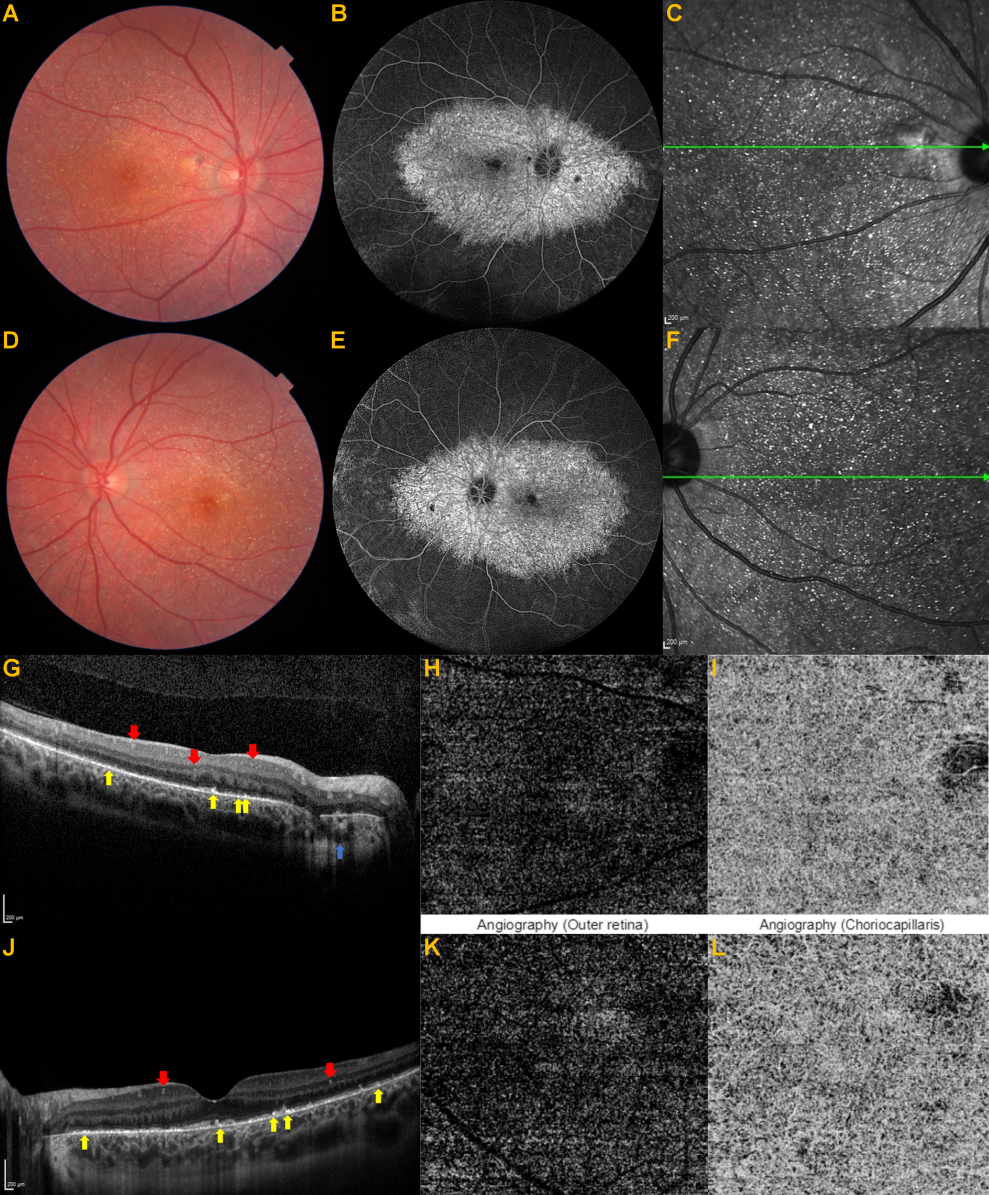
Color fundus pictures (A, right eye and D, left eye); normal optic disc and almost symmetrical widespread refractile white-yellow retinal deposits radiating 360° from posterior pole towards the equator. Venous phase of wide-angle fluorescein angiogram (B, right eye and E, left eye); bilateral circular late staining corresponding to the subtle atrophic areas at the posterior pole. Reflectance images (C, right eye and F, left eye) and enhanced depth optical coherence tomographic sections (G, right eye and J, left eye); red arrows show intraretinal hyperreflective dots, yellow arrows show hyperreflective plaque-like accumulations on the RPE-Bruch’s membrane and blue arrow shows choroidal excavation adjacent to the temporal disc rim in OD. Optical coherence tomography angiography sections outer retina slabs (H, right eye and K, left eye); almost normal outer retina; choriocapillaris slabs (I and L); a few areas of flow deficits.

A 15-year-old, otherwise healthy Caucasian boy was examined with mild visual complaints and very subtle night blindness in July 2020. On ophthalmological examination, visual acuity was 20/25 in both eyes with the correction of (–1.50 –0.50 α 90) in OD and (–1.00 –1.00 α 90) in OS on Snellen chart. Slit-lamp examination exhibited a clear cornea without any corneal crystals and clear lens OU. Fundus examination disclosed a normal looking optic disc and almost symmetrical widespread refractile white-yellow retinal deposits radiating 360° from posterior pole towards the equator bilaterally (Figure 1A/D (Fig. 1)) and there was a slightly pigmented atrophic-looking area adjacent to the temporal disc rim in OD. Fluorescein angiogram (FA) (Heidelberg Spectralis, Heidelberg Engineering, Heidelberg, Germany) revealed bilateral circular-looking late staining corresponding to the subtle atrophic areas at the posterior pole (Figure 1B/E (Fig. 1)). Fundus reflectance image exhibited multiple bright dots surrounding the both foveas (Figure 1C/F (Fig. 1)) and enhanced depth optical coherence tomography (OCT) (Heidelberg Spectralis, Heidelberg Engineering, Heidelberg, Germany) examination revealed a few intraretinal hyperreflective dots and hyperreflective plaque-like accumulations on the RPE-Bruch’s membrane (Figure 1G/J (Fig. 1)). While outer retina slab of optical coherence tomography angiography (OCT-A) (Triton, Topcon Inc., Oakland, New Jersey, USA) was bilaterally almost normal (Figure 1H/K (Fig. 1)), choriocapillaris slabs demonstrated a few areas of flow deficit (Figure 1I/L (Fig. 1)). Based on these findings, a clinical diagnosis of BCD was established and a yearly ophthalmic examination was recommended. 

**Figure 2:**
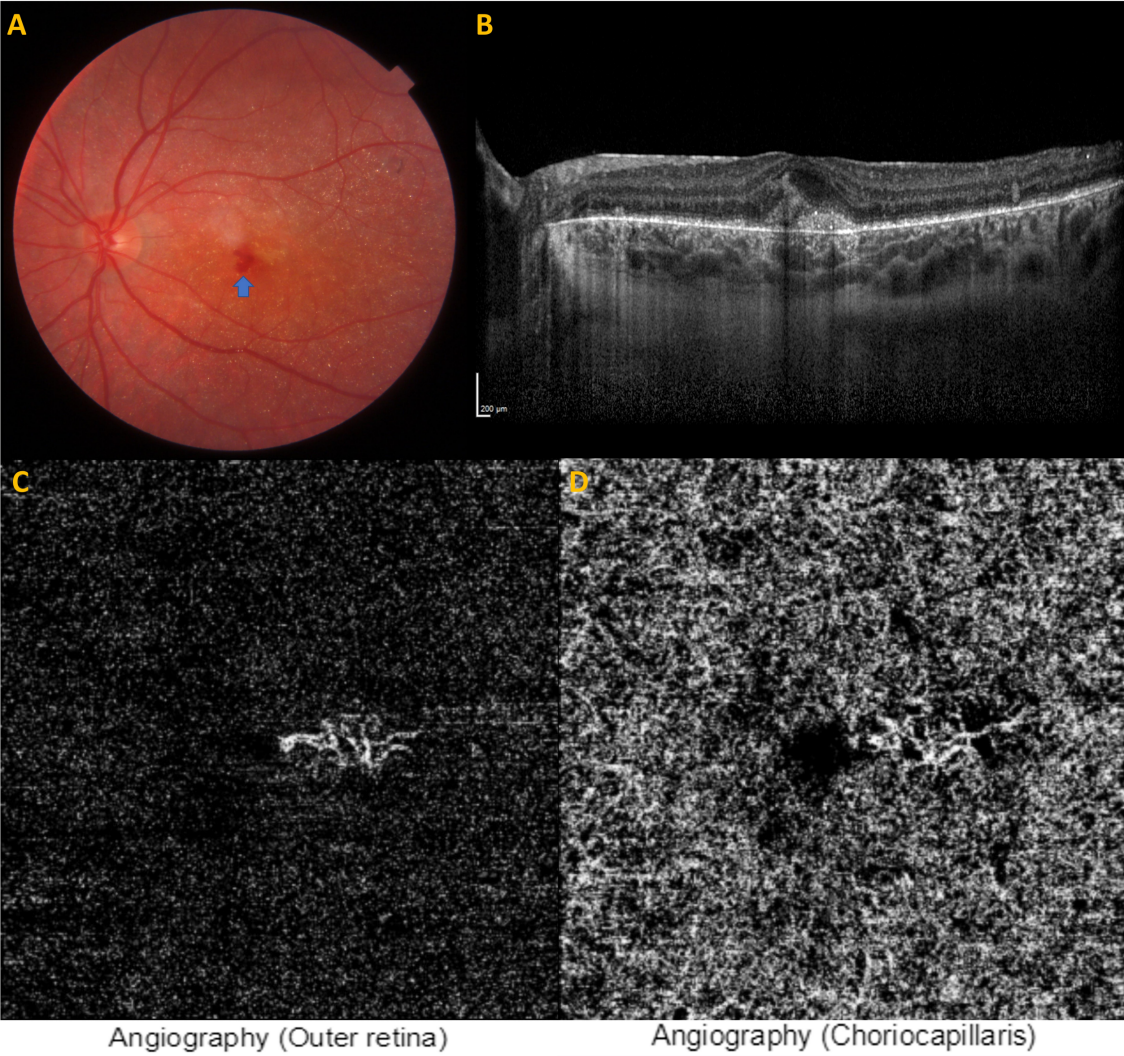
Color fundus picture of the left eye (A), blue arrow shows foveal retinal hemorrhage. Optical coherence tomographic section of the left eye (B), macular neovascularization and intraretinal cysts. Optical coherence tomography angiographic section outer retina slab of the left eye (C), neovascular tuft of vessels. Optical coherence tomography angiographic section choriocapillaris slab of the left eye (D), a few areas of flow deficit.

Unfortunately, genetic analysis and electrophysiological tests could not be performed. Almost a year later, he experienced a sudden visual decrease in his left eye. This time, while the visual acuity of the right eye was unchanged, the best-corrected visual acuity was 20/100 in OS. The appearance of the right fundus was unchanged but there was a significant foveal retinal hemorrhage in OS (Figure 2A (Fig. 2)). There was evidence of a MNV with some intraretinal fluid on OCT (Figure 2B (Fig. 2)). OCT-A confirmed the presence of the neovascular complex (Figure 2C/D (Fig. 2)). FA was not performed as we were confident about the diagnosis.

**Figure 3:**
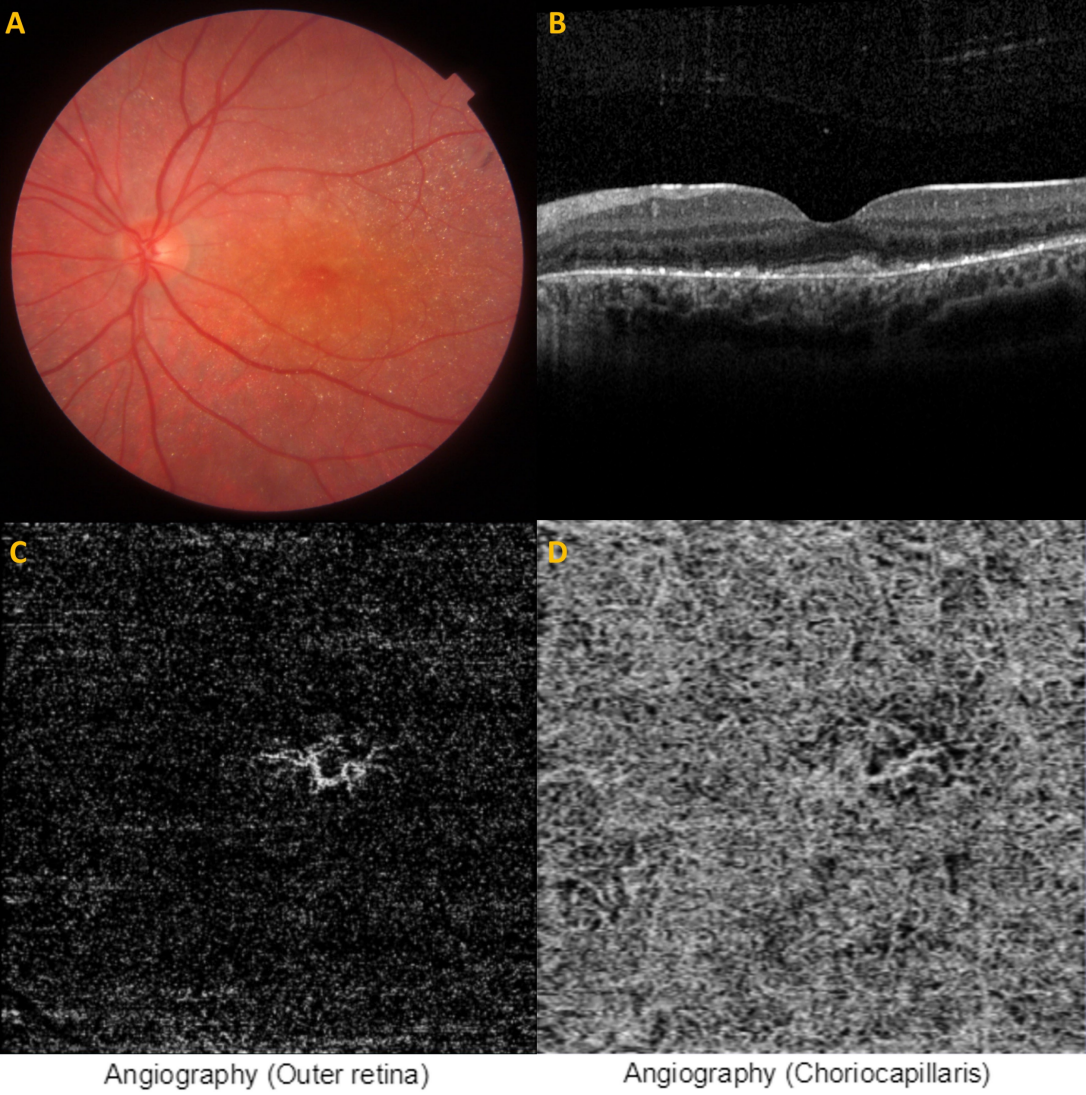
Fifteen months after the intravitreal ranibizumab injection. Color fundus picture of the left eye (A); scattered retinal crystals and almost normalized fovea. Optical coherence tomographic section of the left eye (B), subtle subfoveal neovascularization. Optical coherence tomography angiographic section outer retina slab of the left eye (C), neovascular tuft of vessels. Optical coherence tomography angiographic section choriocapillaris slab of the left eye (D), a few areas of flow deficit.

The therapeutic options were discussed with the parents and the patient, and intravitreal ranibizumab injection was administered without any complication under general anesthesia in OS. Two months later, left visual acuity improved to 20/25 and the intraretinal hemorrhage was almost completely cleaned up but the presence of MNV was still observable on OCT and OCT-A. Regular follow-ups were carried out and 15 months after the injection visual acuity of the left eye was 20/25 with no visual symptoms. OCT and OCT-A illustrated a very stable looking MNV with a relatively diminished lesion size (Figure 3A-D (Fig. 3)).

## Discussion

MNV is a rare clinical finding in children that can cause significant visual impairment if left untreated [14], [15]. Posterior uveitis/post inflammation, retinal dystrophies, ocular trauma, neoplasms, pathological myopia and idiopathic variety are among the causes of pediatric MNVs and retinal dystrophies are present only in 11.5 [16] to 39.5% [17] of the pediatric cases. Best disease is the most common form of dystrophies in association with pediatric MNVs, and there are only anectodal case reports describing the MNV associated with BCD (Table 1 (Tab. 1)). Of those, only two cases with BCD were under 18 years of age [7], [13]. Atmaca et al. [7] reported a 13-year-old girl with BCD who developed a unilateral peripapillary MNV 14 months after the initial diagnosis but no treatment was administered at that time. Gungor Kobat et al. [13] reported a 13-year-old girl with a unilateral foveal scar without any leakage due to a neovascularization. To our best knowledge, the present case was the third pediatric case reported thus far and the first pediatric case which was treated successfully with a single intravitreal ranibizumab injection.

Intravitreal anti-VEGF administration is a viable treatment option in childhood MNVs despite being an off-label treatment and was previously reported in several retrospective clinical studies [14], [16], [17]. However, it is less convenient to perform intravitreal injections in pediatric age group as they can only be given under general anaesthesia as the administration in the present case. On the other hand, fewer anti-VEGF injections seems to be required in children with MNV when compared to adult patients with wet-type age-related macular degeneration [18]. The efficacy and safety of ranibizumab 0.5 mg administration in five adolescent patients aged 13–17 years with any choroidal neovascularization etiology enrolled in the prospective 12 month long Minerva study was analyzed as a subgroup involving a total of 183 patients [15]. These patients received a mean of three injections (range 2–5) and retreatment was warranted only in the presence of disease activity. The patients gained a mean of +16.6 letters at month 12.

We observed the occurrence of a unilateral MNV in a 15-year-old boy just a year after the initial diagnosis of BCD and were able to treat the MNV successfully with a single intravitreal ranibizumab injection.

## Conclusion

Though rare, macular neovascular membrane may compromise the vision further if left untreated in eyes with BCD. The present case is the third reported pediatric case with MNV in association with BCD and the first pediatric BCD patient who received anti-VEGF agent and did anatomically and functionally well after the treatment.

## Notes

### Authors’ ORCIDs

- Mustafa Kayabasi: 0000-0003-2059-0696
- Ferdane Atas: 0000-0002-6743-1142
- Ali Osman Saatci: 0000-0001-6848-7239

### Competing interests

The authors declare that they have no competing interests.

## Figures and Tables

**Table 1:**
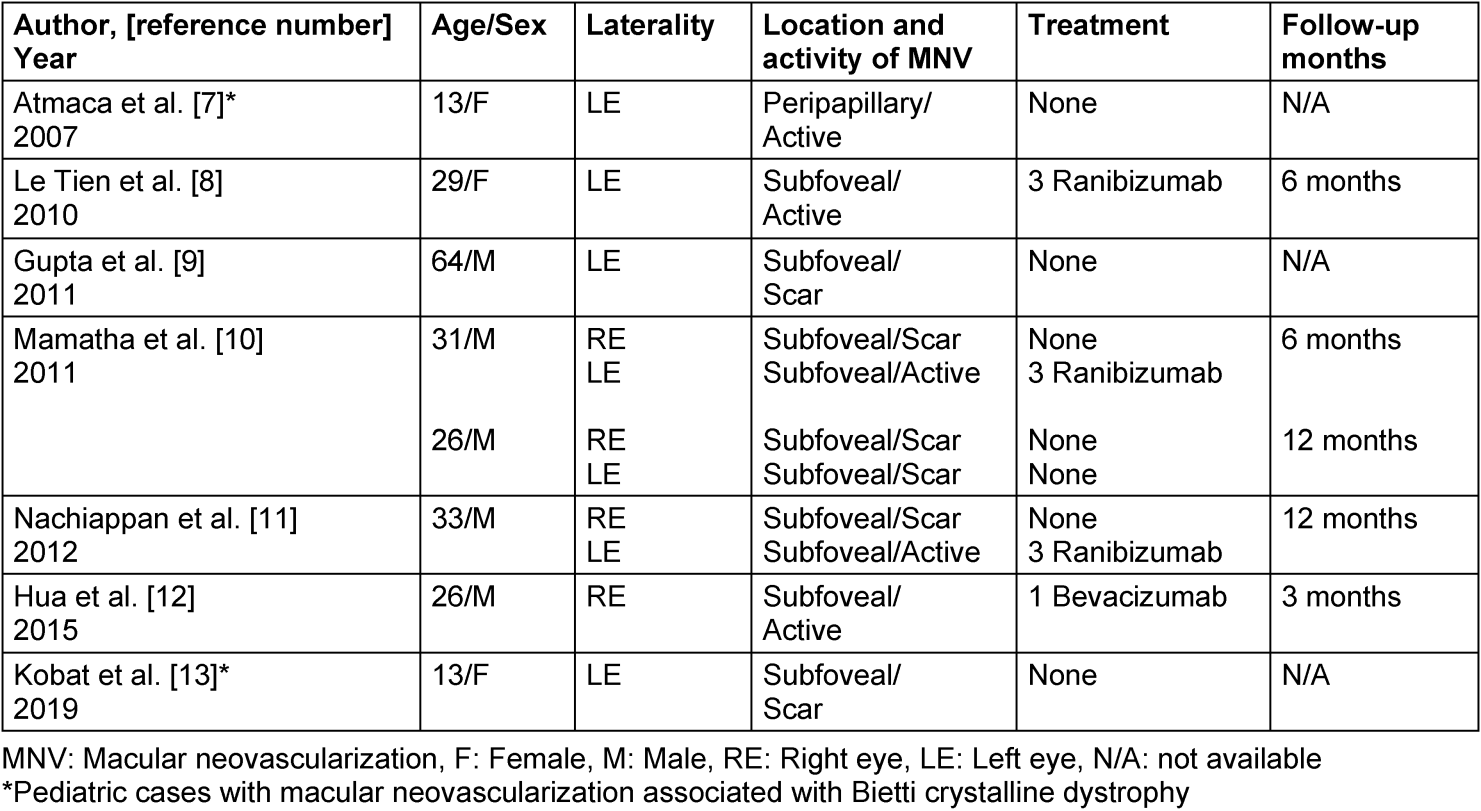
Previous reports on macular neovascularization associated with Bietti crystalline dystrophy

## References

[1] Yuzawa M, Mae Y, Matsui M (1986). Bietti’s crystalline retinopathy. Ophthalmic Paediatr Genet.

[2] Osman Saatci A, Can Doruk H (2014). An overview of rare and unusual clinical features of Bietti’s crystalline dystrophy. Med Hypothesis Discov Innov Ophthalmol.

[3] İpek ŞC, Ayhan Z, Kadayıfçılar S, Saatci AO (2019). Swept-source optical coherence tomography angiography in a patient with Bietti crystalline dystrophy followed for ten years. Turk J Ophthalmol.

[4] Saatci AO, Doruk HC, Yaman A (2014). Cystoid macular edema in Bietti’s crystalline retinopathy. Case Rep Ophthalmol Med.

[5] Padhi TR, Kesarwani S, Jalali S (2011). Bietti crystalline retinal dystrophy with subfoveal neurosensory detachment and congenital tortuosity of retinal vessels: case report. Doc Ophthalmol.

[6] Saatci AO, Yaman A, Berk AT, Söylev MF (1997). Macular hole formation in Bietti’s crystalline retinopathy. A case report. Ophthalmic Genet.

[7] Atmaca LS, Muftuoglu O, Atmaca-Sonmez P (2007). Peripapillary choroidal neovascularization in Bietti crystalline retinopathy. Eye (Lond).

[8] Le Tien V, Atmani K, Querques G, Massamba N, Souied EH (2010). Ranibizumab for subfoveal choroidal neovascularization in Bietti crystalline retinopathy. Eye (Lond).

[9] Gupta B, Parvizi S, Mohamed MD (2011). Bietti crystalline dystrophy and choroidal neovascularisation. Int Ophthalmol.

[10] Mamatha G, Umashankar V, Kasinathan N, Krishnan T, Sathyabaarathi R, Karthiyayini T, Amali J, Rao C, Madhavan J (2011). Molecular screening of the CYP4V2 gene in Bietti crystalline dystrophy that is associated with choroidal neovascularization. Mol Vis.

[11] Nachiappan K, Krishnan T, Madhavan J (2012). Ranibizumab for choroidal neovascular membrane in a rare case of Bietti’s crystalline dystrophy: a case report. Indian J Ophthalmol.

[12] Hua R, Chen K, Hu Y, Wang X, Chen L (2015). Relapse of choroidal neovascularization in Bietti’s crystalline retinopathy following anti-vascular endothelial growth factor therapy: a case report. Exp Ther Med.

[13] Kobat SG, Gul FC, Yusufoglu E (2019). Bietti crystalline dystrophy and choroidal neovascularization in childhood. Int J Ophthalmol.

[14] Barth T, Zeman F, Helbig H, Oberacher-Velten I (2016). Etiology and treatment of choroidal neovascularization in pediatric patients. Eur J Ophthalmol.

[15] Hykin PG, Staurenghi G, Wiedemann P, Wolf S, Liew SHM, Desset-Brethes S, Staines H, Li J, Lai TYY, MINERVA study group (2021). Ranibizumab 0.5 mg treatment in adolescents with choroidal neovascularization: subgroup analysis data from the MINERVA study. Retin Cases Brief Rep.

[16] Ranjan R, Salian R, Verghese S, Manayath GJ, D’Souza P, Kanakath AV, Shah PK, Saravanan VR, Venkatapathy N (2022). Pediatric choroidal neovascularization: etiology and treatment outcomes with anti-vascular endothelial growth factors. Eur J Ophthalmol.

[17] Padhi TR, Anderson BJ, Abbey AM, Yonekawa Y, Stem M, Alam D, Modi RR, Savla LP, Trese MT, Capone A, Drenser KA, Besirli CG (2018). Choroidal neovascular membrane in paediatric patients: clinical characteristics and outcomes. Br J Ophthalmol.

[18] Kohly RP, Muni RH, Kertes PJ, Lam WC (2011). Management of pediatric choroidal neovascular membranes with intravitreal anti-VEGF agents: a retrospective consecutive case series. Can J Ophthalmol.

